# The vital role of citrate buffer in acetone–butanol–ethanol (ABE) fermentation using corn stover and high-efficient product recovery by vapor stripping–vapor permeation (VSVP) process

**DOI:** 10.1186/s13068-016-0566-2

**Published:** 2016-07-19

**Authors:** Chuang Xue, Zixuan Wang, Shudong Wang, Xiaotong Zhang, Lijie Chen, Ying Mu, Fengwu Bai

**Affiliations:** School of Life Science and Biotechnology, Dalian University of Technology, Linggong Road 2, Dalian, 116024 China; School of Life Sciences and Biotechnology, Shanghai Jiao Tong University, Shanghai, 200240 China

**Keywords:** ABE fermentation, Corn stover, Butanol recovery, Vapor stripping–vapor permeation

## Abstract

**Background:**

Butanol is not only an important solvent and chemical intermediate in food and pharmaceutical industries, but also considered as an advanced biofuel. Recently, there have been resurging interests in producing biobutanol especially using low-cost lignocellulosic biomass, but the process still suffers from low titer and productivity. The challenge for the bioconversion approach is to find an effective way of degrading materials into simple sugars that can then be converted into fuels by microorganisms. The pretreatment of lignocellulosic biomass is the great important process in influencing butanol production and recovery, finally determining its eco-feasibility in commercialization.

**Results:**

The effects of various strengths of citrate buffer on enzymatic hydrolysis and acetone–butanol–ethanol fermentation using corn stover or glucose as feedstock were investigated. The strengths of citrate buffer in the range of 20–100 mM had no effect on enzymatic hydrolysis, but greatly influenced the performance of ABE fermentation using corn stover hydrolysate. When 30 mM citrate buffer was used for enzymatic hydrolysis, the fermentation broth with the maximum butanol and ABE concentrations of 11.2 and 19.8 g/L were obtained from 30.9 g/L glucose and 9.7 g/L xylose, respectively, which was concentrated to 100.4 g/L butanol and 153.5 g/L ABE by vapor stripping–vapor permeation process. Furthermore, using glucose as sole carbon source, there were no cell growth and ABE production in the P2 medium with 80 or 100 mM citrate buffer, indicating that higher concentrations of citrate buffer had deleterious effect on cell growth and metabolism due to the variation of cells internal pH and cell membrane permeability. To mimic in situ product recovery for ABE fermentation, the VSVP process produced the condensate containing 212.0–232.0 g/L butanol (306.6–356.1 g/L ABE) from fermentation broth containing ~10 g/L butanol (~17 g/L ABE), the performance of which was more effective than pervaporation and gas stripping.

**Conclusions:**

As it has significant impact on butanol fermentation, the strength of citrate buffer is of great importance in lignocellulosic butanol fermentation. Compared with pervaporation and gas stripping, the VSVP process has great potential for efficient butanol recovery in biobutanol production.

## Background

Butanol as an important chemical and potential fuel could be produced via ABE fermentation using maize, sugar cane, etc., but the use of these food-related feedstocks to produce biofuel may not be a sustainable solution to world’s energy needs [1, 2]. Agriculture-derived lignocellulose biomass is a promising alternative for sustainable production of biofuels, with many advantages such as renewability, low cost and abundance at a global scale. Corn stover is one of the most abundant agricultural residues in China, the rational utilization of which could reduce smog pollution due to its burning in rural areas. More importantly, bioconversion of corn stover to transportation fuel could provide an environmentally friendly route to utilize the agricultural residues and boost rural economy [2, 3].

Lignocellulose consists mainly of cellulose, hemicellulose and lignin, and is highly recalcitrant to microbial degradation due to its high cellulose crystallinity and complex cross-linking structure [4]. To make it available for butanol fermentation, lignocellulosic biomass is required to be pretreated and enzymatic hydrolyzed into fermentable sugars for butanol-producing microorganisms. To date, various pretreatment approaches have been intensively developed to facilitate the enzymatic hydrolysis of corn stover, such as chemical pretreatments (e.g., alkali pretreatment, acid pretreatment, ionic liquids pretreatment) and physico-chemical pretreatments (e.g., steam explosion, liquid hot water, ammonia fiber explosion) [5]. In enzymatic hydrolysis, the use of buffer salts is of great importance to maintain cellulase activity for release of more fermentable sugars, and thus influencing butanol fermentation and commercially feasibility. Enzymes are optimally active at a specific pH and temperature to achieve the maximum hydrolysis of substrates [4]. For the optimal cellulase activity, the enzyme reaction proceeds at commonly prepared conditions, such as pH 4.8 and 50 mM citrate buffer [6–8]. There was rare study to consider whether this designated condition for enzymatic hydrolysis is also optimal for subsequent butanol fermentation. Furthermore, carrying out enzyme hydrolysis at 50 mM citrate buffer strength is not commercially feasible when the process was scaled up at industrial level. Therefore, it could be crucial to determine if enzymatic hydrolysis could be conducted in a lower strength buffer, simultaneously with improving biobutanol production.

Even though lots of efforts have been made by engineering *Clostridium* spp. and heterogenous strains, butanol concentration in fermentation broth could not exceed 2 % (w/v) [1]. Distillation is nowadays the most popular technique used in industry for ABE product recovery. However, this technique has several disadvantages such as high investment costs, low selectivity and high energy consumption [9]. During *n*-butanol recovery by distillation, most of the energy consumption originates from the evaporation of the water in the feed, which is considered to be an energy-intensive process. Therefore, several other techniques such as gas stripping, liquid–liquid extraction, pervaporation and adsorption, etc., have received increasing attention as they could continuously remove ABE solvents from fermentation broth and reduce the inhibition of ABE to cells by integrating with ABE fermentation [1, 10]. Among them, pervaporation is the membrane-based technology and considered as an energy-efficient alternative to distillation for removing solvents from the dilute fermentation broth. Numerous studies on butanol recovery by pervaporation have been conducted using membranes fabricated by various materials [11]. Since the ABE solvents or fermentation broth directly contact with one side of the membrane during butanol recovery, the main concern of pervaporation process is that the membranes tend to be contaminated by the adsorption and infiltration of cells and nutrients from fermentation broth. The vapor stripping–vapor permeation (VSVP) process, termed membrane-assisted vapor stripping was more rarely studied than pervaporation for butanol recovery, which could prevent membrane fouling due to volatilized organic compounds contacting both sides of the membrane during mass transfer. Furthermore, it was reported that the VSVP process was at least 65 % more energy efficient than conventional distillation approaches [12]. Till now, there is no study on VSVP process for recovering butanol derived from agricultural residues such as corn stover.

The goal of this study was to determine the effect of various strengths of citrate buffer on enzymatic hydrolysis and ABE fermentation using corn stover or glucose as feedstock. The VSVP process was demonstrated to recover ABE solvents and mimic in situ product recovery from fermentation broth during ABE fermentation using corn stover. Furthermore, the performance of VSVP process, pervaporation and gas stripping were compared to elucidate the potential of VSVP process for butanol recovery in biobutanol production.

## Results and discussion

### The effect of citrate buffer on enzymatic hydrolysis and ABE fermentation

The composition of raw corn stover used in this work contained 41.2 % cellulose, 21.6 % hemicellulose, 32.7 % lignin and ash. After 2 % NaOH pretreatment, the solid residues varied to contain 62.2 % cellulose, 24.6 % hemicellulose, 11.3 % lignin and ash, with weight loss of ~40 %. The weight loss of corn stover in alkali pretreatment was mainly attributed to the solubilization of components in corn stover such as lignin, hemicellulose and other soluble extractants. Thus, ~90 % of cellulose was recovered in alkali pretreatment, but ~80 % of lignin was removed from corn stover due to the solubilization of lignin in NaOH solution. Compared with dilute H_2_SO_4_, lime and NH_3_/HCl pretreatment, pretreatment of corn stover with 2 % NaOH was proved to substantially increase the lignin removal and improve the accessibility and digestibility of cellulose [6]. Furthermore, the enzymatic hydrolysate from NaOH-pretreated corn stover contained higher content of fermentable sugars and less inhibitors. Compared with dilute acid and steam explosion pretreatment, alkali pretreatment only produced acetic acid in the liquid stream, but dilute acid and steam explosion produced inhibitors (furfural, HMF, acetic acid and formic acid) soluble in the liquid stream [13]. The furfural and HMF existed in fermentation broth were considered as inhibitors in microorganism fermentation. Therefore, the hydrolysate from alkali pretreatment may be the most favorable carbon source for microorganism.

The sodium citrate buffers with different concentrations were used to investigate the effect on enzymatic hydrolysis and ABE fermentation using corn stover. When 10 g of corn stover solid residues from alkali pretreatment was added into 100 mL sodium citrate buffers with the concentration ranges of 20–100 mM, respectively, 81.8 ± 2.3 g/L total sugars (54.0 ± 1.5 g/L glucose, 18.8 ± 0.5 g/L xylose, 7.5 ± 0.7 g/L cellobiose, 1.7 ± 0.1 g/L arabinose) were released in the enzymatic hydrolysis (see Table 1). There were no prominent differences in sugars concentrations released from corn stover when using different strengths of the citrate buffer. The one-way ANOVA analysis indicated that citrate buffers in the test concentrations had no significant effect on fermentable sugars released from corn stover due to *P* value of >0.05. After inoculation of the seed and addition of other P2 medium components, the corn stover hydrolysates with various citrate strengths were used for ABE fermentations, with initial total sugars of 72.2 ± 2.8 g/L (glucose 46.1 ± 1.0 g/L, xylose 17.8 ± 1.0 g/L, cellobiose 6.1 ± 0.5 g/L, arabinose 1.5 ± 0.1 g/L, respectively). When sodium citrate concentrations in the hydrolysate increased from 20 to 30 mM, butanol and ABE titer increased from 9.4 and 15.8 to 11.2 and 19.7 g/L, respectively, but then gradually decreased to 6.4 and 11.0 g/L when sodium citrate in the hydrolysate increased to 100 mM. The maximum butanol concentration, yield and productivity were obtained with 11.2 g/L, 0.28 g/g, 0.19 g/L/h, respectively, when 30 mM citrate buffer was used for enzymatic hydrolysis. Compared with other buffer strengths, more glucose were consumed in ABE fermentation under the scenario with 30 mM citrate buffer. There was no decrease of cellobiose and arabinose concentrations in fermentation broth as *Clostridium beijerinckii* CC101 could not utilize them as carbon sources. The one-way ANOVA analysis indicated that citrate buffers had very significant effect on butanol production as *P* value was less than 0.001.

**Table 1:** The performance of enzymatic hydrolysis and ABE fermentation under various citrate buffer strengths using corn stover

	Sodium citrate buffer strengths (mM)							
	20	30	40	60	80	100	100 (dilute)	30 (dilute)
Initial glucose, g/L	45.7	45.1	46.8	47.4	47.1	46.1	23.5	23.8
Initial xylose, g/L	17.9	18.4	18.5	18.0	18.6	20.5	9.9	9.8
Initial cellobiose, g/L	6.6	6.3	6.7	6.5	6.4	6.9	2.7	3.7
Initial arabinose, g/L	1.8	1.5	2.8	2.1	1.5	1.5	0.9	0.8
Residual glucose, g/L	19.0	14.2	16.9	19.6	26.0	27.0	2.2	0.2
Residual xylose, g/L	6.5	8.7	11.2	7.5	8.1	8.1	2.6	2.2
Maximum OD	2.4	2.9	2.6	1.9	1.3	1.0	1.9	2.6
Fermentation time, h	60	60	60	60	60	60	48	48
Butanol, g/L	9.4	11.2	8.2	8.0	7.1	6.4	6.5	6.1
Acetone, g/L	6.0	7.5	5.0	5.0	4.7	4.4	3.3	3.9
Ethanol, g/L	0.4	1.1	0.4	0.4	0.3	0.3	0.2	0.2
Total ABE, g/L	15.8	19.8	13.6	13.4	12.1	11.1	10.0	10.2
Butanol yield, g/g	0.25	0.28	0.22	0.21	0.22	0.20	0.23	0.20
Butanol productivity, g/L/h	0.16	0.19	0.14	0.13	0.12	0.11	0.14	0.13
Acetic acid, g/L	2.4	3.6	2.8	3.6	2.6	1.9	2.4	3.0
Butyric acid, g/L	3.6	0.3	2.6	1.0	0.4	0.6	0.6	1.9

The butanol yield was calculated based on total consumption of glucose and xylose

**Fig. 1:**
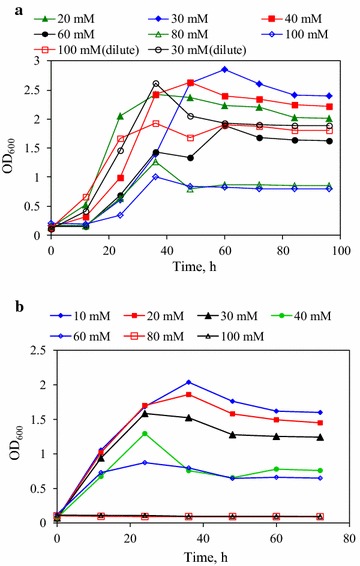
Kinetics of cell growth in ABE fermentation in various strengths of citrate buffer. The strengths of citrate buffer are in the range of 10–100 mM. **a** Corn stover hydrolysate as carbon source in citrate buffer with different concentrations (20, 30, 40, 60, 80, 100 mM); **b** glucose as carbon source in citrate buffer with different concentrations (10, 20, 30, 40, 60, 80, 100 mM)

Kinetics of cell growth in ABE fermentation in various strengths of citrate buffer is shown in Fig. 1. The maximum cell growth was obtained in the corn stover hydrolysate medium with 30 mM citrate buffer. The growth of *C. beijerinckii* CC101 was deterred by the hydrolysate medium with more than 50 mM citrate buffer. Higher citrate strengths inhibited cell growth by reducing the cells internal pH and proton motive force, and changing cell membrane permeability [14]. Higher strength of citrate buffer will lead to higher concentration of undissociated citric acid and higher medium osmolality, which can directly affect cell growth. In addition, citrate buffers may chelate trace elements, which may influence the optimum cell growth in the medium [8, 15]. Since *P* value was between 0.05 and 0.001, citrate buffers had significant effect on cell growth in corn stover hydrolysate. To further verify the inhibitory effect of citrate buffer on cell growth and butanol production, the corn stover mediums with 30 and 100 mM citrate buffer were diluted twice and then used for ABE fermentation. As shown in Fig. 1 and Table 1, the cell growths were enhanced in these two diluted mediums. Compared with corn stover medium with 100 mM citrate buffer, the dilute medium was more effective for cell growth, with the maximum OD and butanol productivity increased by 90 and 27.3 %, respectively.

To investigate the effect of citrate buffer on ABE fermentation, the sodium citrate buffers with the concentrations of 10–100 mM were added to P2 medium using initial glucose of 66.0 ± 2.0 g/L as sole carbon source, respectively. As shown in Table 2, when sodium citrate concentration increased from 10 to 60 mM, butanol and ABE concentration decreased gradually from 9.1 and 14.2 to 4.6 and 7.3 g/L in the time course of 48 h, respectively. When the sodium citrate concentrations were at 80 and 100 mM, there were no glucose consumption and ABE production in fermentation broth. As *P* value was less than 0.001, the strength of citrate buffer had very significant effect on butanol production in glucose P2 medium. As shown in Fig. 1b, the cell growth gradually decreased with the increase of citrate buffer strength, and there were no cell growth at 80 and 100 mM of citrate buffer. The one-way ANOVA analysis indicated that citrate buffer had very significant effect on cell growth in glucose P2 medium as *P* value was much less than 0.001. Furthermore, estimated by *P* value, the effect of citrate buffer on cell growth in glucose P2 medium was more significant than that in corn stover hydrolysate P2 medium. When using glucose as sole carbon source in the P2 medium, sodium citrate had strong toxic effect on cell growth and ABE production. But, when using corn stover hydrolysate, the extractants from corn stover as well as cellulose cocktail enzymes may interact with citrate buffer and alleviate the effect of citrate buffer on cell growth.

**Table 2:** The performance of ABE fermentation with various citrate buffer strengths using glucose

	Sodium citrate buffer strengths (mM)						
	10	20	30	40	60	80	100
Initial glucose, g/L	67.0	68.0	65.0	64.0	66.0	66.0	68.0
Residual glucose, g/L	29.0	32.0	32.0	43.0	43.0	65.0	68.0
Maximum OD	2.0	1.9	1.6	1.3	0.9	0.1	0.1
Fermentation time, h	48	48	36	24	24	0	0
Butanol, g/L	9.0	8.3	6.8	4.3	4.5	0	0
Acetone, g/L	4.9	4.0	3.1	2.4	2.6	0	0
Ethanol, g/L	0.2	0.2	0.2	0.1	0.1	0	0
Total ABE, g/L	14.1	12.5	10.1	6.8	7.2	0	0
Butanol yield, g/g	0.24	0.23	0.21	0.20	0.20	0	0
Butanol productivity, g/L/h	0.19	0.17	0.14	0.13	0.12	0	0
Acetic acid, g/L	1.8	2.3	3.2	3.2	3.2	0	0
Butyric acid, g/L	2.1	1.8	1.3	1.1	0.8	0	0

### Product recovery from fermentation broth by vapor stripping–vapor permeation process

**Fig. 2:**
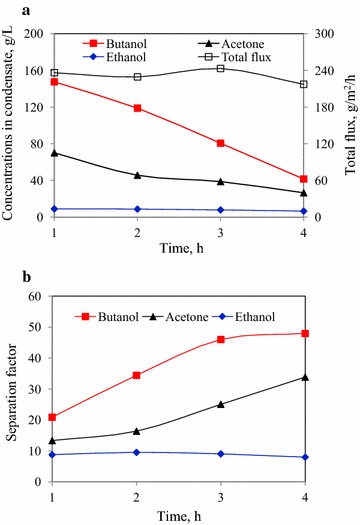
The performance of ABE recovery from fermentation broth using vapor stripping–vapor permeation process. **a** ABE concentrations in condensate and total flux; **b** separation factors of butanol, acetone and ethanol

The vapor stripping–vapor permeation (VSVP) process with the pure PDMS membrane was carried out to investigate the performance of product recovery from active fermentation broth. The VSVP process is the membrane-based technology in which the solvent mixture vaporizes by gas stripping and then contacts with one side of the membrane. The vapor mixture is diffused into the membrane, then desorbed to the permeate side as vapor under vacuum. Finally, the vapor is condensed at a low temperature. The fermentation broth containing 11.2 g/L butanol, 7.5 g/L acetone and 1.1 g/L ethanol in 75 mL was used in vapor stripping–vapor permeation process, which was derived from corn stover hydrolysate treated with 30 mM citrate buffer in the enzymatic pretreatment (see Table 1). When vapor stripping–vapor permeation experiment was carried out in 4 h, ~90 % of ABE solvent could be recovered from fermentation broth. The butanol, acetone and ethanol concentration in fermentation broth decreased from 11.2, 7.5 and 1.1 to 0.7, 0.6 and 0.8 g/L, respectively. The recovery rate of butanol and ABE were 93.7 and 89.4 %, respectively. In the first hour of product recovery process, the condensate containing 147.5 g/L butanol, 70.0 g/L acetone and 8.8 g/L ethanol was achieved, with totally 236.2 g/L ABE solvents (see Fig. 2). Then, the butanol, acetone and ethanol concentration in the condensate gradually decreased to 41.4, 26.4 and 6.4 g/L at 4 h, respectively. The total flux was relatively stable in the range of 217.2–243.1 g/m^2^/h, while butanol flux decreased with time due to the decreased butanol concentration in fermentation broth. The separation factors of butanol and acetone increased with time, indicating that the selectivities of butanol and acetone over water in VSVP process were higher in the feed with low butanol and acetone concentrations. There was no variation of ethanol separation factor as the ethanol concentration in feed solution maintained stable at a low level. The average butanol and ABE concentrations were 100.4 and 153.5 g/L, respectively. The average separation factors of butanol, acetone and ethanol were 34.2, 13.9 and 8.1, respectively. The demonstrating results showed that the VSVP process was very effective for ABE solvents recovery from fermentation broth.

### Simulation of in situ product recovery during ABE fermentation

To mimic in situ product recovery during ABE fermentation, the vapor stripping–vapor permeation process was conducted using fermentation broth with butanol, acetone and ethanol concentrations of 9.7, 4.9 and 2.3 g/L at 37 °C. According to our previous studies, in situ product recovery during ABE fermentation are usually conducted at ~10 g/L butanol in fermentation broth, which not only could alleviate butanol toxicity to cells but also obtain a high butanol concentration recovered in condensate [16]. Therefore, the fermentation broth containing ~10 g/L butanol in 500 mL was used to investigate the performance of VSVP process to continuously recover ABE solvents.

Since the volume of feed solution was much greater than the recovered volume per hour, the performance of the VSVP process was very stable due to the ABE concentrations in feed solution maintained at stable level. When butanol, acetone and ethanol concentrations in feed solution were in the range of 9.4–9.7, 4.5–4.7 and 2.1–2.3 g/L, respectively, the VSVP process produced the recovered condensate containing 212.0–232.0 g/L butanol, 86.3–115.5 g/L acetone and 8.3–8.6 g/L ethanol, with total flux of 117.2–124.1 g/m^2^/h (see Table 3). The average separation factors of butanol, acetone and ethanol were 29.8, 24.3 and 3.9, respectively.

**Table 3:** The comparison of ABE recovery by VSVP process, pervaporation and gas stripping

	ABE concentrations in condensate (g/L)			Total flux (g/m^2^/h)	Apparent stripping rate (g/L/h)	SF_B_/SF_A_	Separation factor		
	B	A	E				B	A	E
VSVP	212.0–232.0	86.3–115.5	8.3–8.6	117.2–124.1	1.36–1.44	1.23	29.8	24.3	3.9
Pervaporation	71.5–77.4	35.0–39.8	6.3–6.7	48.8–54.3	0.57–0.63	0.99	8.2	8.3	3.2
Gas stripping	99.8–106.5	41.1–46.3	7.4–7.7	–	1.10–1.20	1.09	10.9	10.0	3.8

The feed solution contained 9.7 g/L butanol, 4.9 g/L acetone and 2.3 g/L ethanol. B, A and E represent butanol, acetone and ethanol, respectively

However, using the same PDMS membrane and feed solution, pervaporation only produced 71.5–77.4 g/L butanol, 35.0–39.8 g/L acetone and 6.3–6.7 g/L ethanol in the condensate, with total flux of 48.8–54.3 g/m^2^/h (see Table 3). The average separation factors of butanol, acetone and ethanol were 8.2, 8.3 and 3.2, respectively. According to our previous studies, the separation factors of homogeneous PDMS membranes with different thicknesses were 7.5–8.0 for butanol recovery at 37 °C, which was enhanced to ~20 when the temperature increased to 80 °C [17]. It was also reported that the PDMS membrane by pervaporation coupling with ABE fermentation was used to recover butanol from fermentation broth, with butanol separation factor of 7.0–10.3 [18]. Therefore, from butanol purity point of view, it should be noted that the VSVP process for butanol recovery is more effective than pervaporation. In addition, hydrophobic fillers or layer composited with the PDMS membrane could improve the separation factor of butanol and mass flux in pervaporation [19, 20]. Therefore, the performance of the VSVP process could be dramatically enhanced if using the composite PDMS membrane.

Gas stripping is an alternative technique that allows the selective removal of volatile solvents from fermentation broth, with solvents recovery from the vapor phase by condensation in a cold trap or via a molecular sieve. The main difference of VSVP process with gas stripping is that the stripped volatile solvents from the vapor phase permeate through a membrane before condensation in a cold trap. When the same fermentation broth with butanol, acetone and ethanol concentrations of 9.7, 4.9 and 2.3 g/L at 37 °C was used as feed, gas stripping produced the condensate containing 99.8–106.5 g/L butanol, 41.1–46.3 g/L acetone and 7.4–7.7 g/L ethanol (see Table 3). The separation factors of butanol, acetone and ethanol were 10.9, 10.0 and 3.8, respectively. In comparison with the VSVP and gas stripping, the stripping rates from fermentation broth in two processes should be theoretically equal if all of the stripped solvents could be recovered and condensed. But the apparent stripping rate of VSVP process could be limited by the membrane performance. If the stripped solvents could not be completely recovered by the membrane, some of stripped solvents and water would circulate back to fermentation broth. Thus, the VSVP process has a lower apparent stripping rate than gas stripping. In present study, the flow rate of stripping gas in the VSVP process is twofold higher than that in gas stripping, the stripping rate of the VSVP process is supposed to be twofold higher than gas stripping. But due to limitation of membrane flux, some of stripped solvents circulated back to fermentation broth, and finally the apparent stripping rate of the VSVP process is <2-fold higher than that of gas stripping. The apparent stripping rate could be enhanced by increasing the membrane area or performance. In addition, the butanol selectivities over acetone (SF_B_/SF_A_) in the three processes are also shown in Table 3. It should be noted that the VSVP process has the highest butanol selectivity over acetone. Therefore, the VSVP process has the best performance for ABE solvents recovery among these three processes. The separation factors of butanol and acetone in VSVP process are at least two times higher than those in pervaporation and gas stripping.

Fermentative production of butanol is limited to low concentration of typically less than 2 % (w/v) butanol, which leads to high separation energy demand by conventional distillation approaches. The process simulation of hybrid vapor stripping–vapor permeation (membrane-assisted vapor stripping system, MAVS) indicated that significant reductions in energy demand are possible for MAVS systems compared with conventional distillation systems to separate ABE solvents from butanol/water binary solutions and ABE/water solutions. The MAVS system is estimated to require 6.2 MJ-fuel/kg-butanol to produce 99.5 % (w/v) butanol from a 1 % (w/v) butanol feed solution, with energy saving of 63 % relative to a benchmark distillation/decanter system [21]. Furthermore, the MAVS pilot unit shows an excellent demonstration that the energy usage of 10.4 MJ-fuel/kg-butanol is required to achieve 85 % butanol recovery from a 1.3 % (w/v) solution [22]. The energy usage could be further reduced by more heat-integrated design. Many scholars invested their attention on studies of membrane-assisted pervaporation for butanol recovery. The butanol vapor stripped from the feed is more concentrated during the stripping process, and the concentrated vapor contacting with one side of the membrane in the VSVP process could dramatically improve the separation factor of butanol. Furthermore, when butanol fermentation was integrated with pervaporation, the membranes tend to be contaminated when in contact with fermentation broth for pervaporation. The cells and macromolecules in the fermentation broth tended to adsorb on or infiltrate into the membrane, which induced the membrane fouling. Membrane fouling significantly increases the downtime and running cost, even though the membrane could be easily recovered by water rinsing. In the VSVP process, the volatile vapor containing ABE solvents and water contacts with both sides of the membrane, which could not induce the membrane contamination. The cells and macromolecules would be detained in the fermentation broth and have no chance to contact with the membrane because they are nonvolatile. Therefore, the VSVP process coupling with ABE fermentation has potential application in the industrial production of biobutanol for long duration.

## Conclusions

To pursue maximum butanol production, the effect of citrate buffer on enzymatic hydrolysis as well as ABE fermentation was investigated as a significant factor in influencing butanol production and cell growth. Different from enzyme reaction that proceeded at commonly prepared condition of 50 mM citrate buffer, the optimal strength of citrate buffer was 30 mM with the maximum butanol (ABE) production of 11.2 g/L (19.8 g/L), making biobutanol production from corn stover more economical. The higher concentration of citrate buffer had strong inhibitory effect on cell growth and ABE fermentation. Compared with pervaporation and gas stripping, the VSVP process was the most efficient technique to produce the condensate containing 212.0–232.0 g/L butanol from fermentation broth with ~10 g/L butanol. The VSVP process had higher butanol separation factor than those in pervaporation and gas stripping in the same scenario, which also had great advantage on preventing the membrane fouling. It is thus desirable to use the VSVP process for efficient butanol recovery in biobutanol production using lignocellulosic biomass.

## Methods

### The pretreatment of corn stover

Corn stover obtained from a local farmer (Zibo City, Shandong Province, China) was air dried, and then ground to 40 meshes followed by suspending in 2 % NaOH solution at 121 °C for 30 min. The solid residues mainly containing cellulose and hemicellulose were washed and filtrated to neutral pH, and then dried at 50 °C to constant weight. Afterwards, the solid residues were hydrolyzed using cellulose enzyme (Novozymes Biotechnology Co., Ltd, Tianjin, China) with ~20 filter paper units (FPU)/g-substrate at 50 °C and 72 h in the buffer. At last, the hydrolyzed corn stover solution was centrifuged at 6000×*g* for 5 min to remove sediments followed by adjusting pH to 6.2 using ammonia, and then stored at 4 °C until being used for the subsequent fermentation. To investigate the effect of the sodium citrate buffer on enzymatic hydrolysis and ABE fermentation, the citrate buffer with the strengths of 10–100 mM were used in enzymatic hydrolysis for corn stover and then corn stover hydrolysate was used for ABE fermentation. Compositional analysis of corn stover and NaOH-pretreated corn stover were performed following National Renewable Energy Laboratory (NREL) protocol [23].

### Culture and media

*Clostridium beijerinckii* CC101, an adaptive mutant strain of *C. beijerinckii* NCIMB 8052 (ATCC 51743) obtained by adaption in a fibrous bed bioreactor were used for ABE fermentation [24]. The seed culture was prepared according to the procedures described previously [24]. The actively grown *C*. *beijerinckii* CC101 cells were incubated at 5 % (v/v) and 37 °C with no agitation.

### ABE fermentation in serum bottles

ABE fermentation was carried out with the P2 medium containing a carbon source (glucose or corn stover hydrolysate), and other components was described previously [16]. The serum bottles each containing 75 mL medium were sterilized by autoclaving at 121 °C and 15 psig for 30 min. All solutions were purged with nitrogen for 1 h through a sterile 0.2 μm filter, either before or after autoclaving. To compare with the effect of citrate buffer on ABE fermentation, citrate buffers in the range of 10–100 mM were also added into the P2 medium with glucose as carbon source, respectively. The culture bottles, tips and tubes, etc., were purchased from Guangzhou Jet Bio-Filtration Co., Ltd.

### Preparation of the homogeneous PDMS membrane

The base solution from the Sylgard^®^184 silicone elastomer kit (Dow Corning, USA) was mixed with the curing agent in the ratio of 10:1 using pentane as the solvent to dilute the mixture. The mixture was stirred completely for 5 min and then 8000×*g* centrifuged for 5 min to wipe off air bubble. The mixture was placed on a cleaning glass plate and cast evenly using a micron film applicator (Paul N. Gardner Company, USA). The mixture on the glass plate was then heated in oven for 3 h at 100 °C. After the membrane cure, the membrane was carefully peeled off for pervaporation. The thickness and effective area of the PDMS membrane were 100 μm and 58 cm^2^, respectively.

### Vapor stripping–vapor permeation for product recovery with the PDMS membrane

**Fig. 3:**
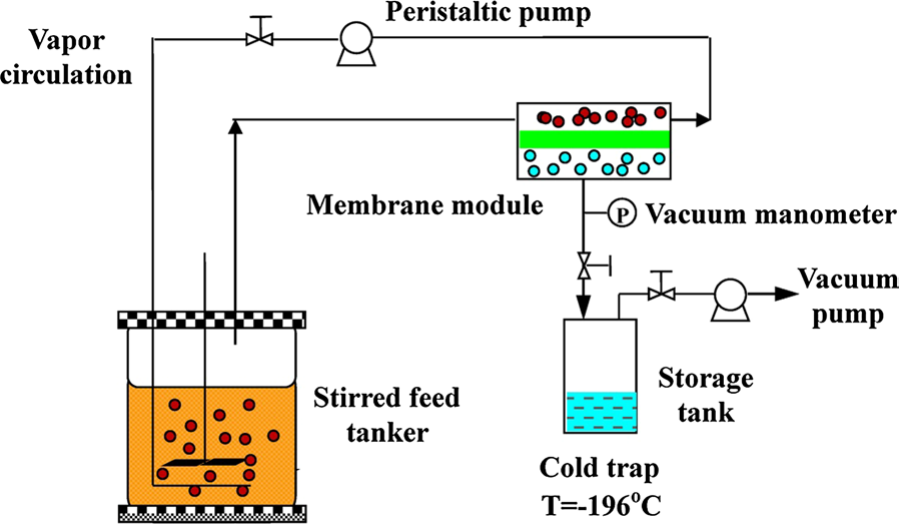
Experimental setup for butanol recovery with vapor stripping–vapor permeation process

The fermentation broth in 75 mL derived from corn stover treated in 30 mM citrate buffer during enzymatic hydrolysis was used to investigate the performance of ABE recovery by vapor stripping–vapor permeation process. The close-circulating vapor stripping–vapor permeation system is illustrated in Fig. 3. The stripping gas at a flow rate of 3.2 L/min was circulated between stirred feed tank and membrane module. Vacuum was provided on the downstream side of the membrane using a vacuum pump with <100 Pa as the driving force. The recovered permeate was collected in the storage tank immersed in liquid nitrogen.

To mimic in situ product recovery during ABE fermentation, the VSVP process was carried out using ABE fermentation broth with 500 mL at 37 °C. The fermentation broth contained 9.7 g/L butanol, 4.9 g/L acetone and 2.3 g/L ethanol. The pervaporation, gas stripping and VSVP processes were compared to recover ABE solvents from the fermentation broth above.

The flux (ABE and total) and separation factor (SF) were calculated as follows:

$$ {\text{Flux}} = \frac{W}{At}, $$

$$ {\text{SF}} = \tfrac{y/(1 - y)}{x/(1 - x)}, $$

where *W* is the weight of the recovered permeate in gram, *A* is the membrane area in m^2^ and *t* is the time (h) for the sample collection. *x* and *y* are the weight fractions of components in the feed and permeate samples in the pervaporation, respectively.

### Gas stripping and pervaporation with the PDMS membrane

To compare with VSVP process, the fermentation broth containing 9.7 g/L butanol, 4.9 g/L acetone and 2.3 g/L ethanol in 500 mL was used to investigate the performance of gas stripping and pervaporation for ABE recovery at 37 °C, respectively. The feed fermentation broth for pervaporation was circulated at a flow rate of 2.0 L/min to minimize the boundary layer thickness and maximize mass transfer. Vacuum was provided on the downstream side of the membrane using a vacuum pump with <100 Pa. The permeate was collected in the storage tank immersed in liquid nitrogen. The close-circulating gas stripping system was described in our previous study [16]. The stripping gas at 1.5 L/min was sparged into the fermentation broth, and then passed through the condenser at ~2 °C for vapor condensation.

### Analytical methods and statistical analysis

The cell density (OD_620_), glucose, butanol, acetone, ethanol, acetic acid and butyric acid were determined according to our previous study [17]. Various sugars in corn stover hydrolysate were analyzed using the HPLC system (Waters 1525) equipped with the column (Aminex HPX-87H, 300 mm × 7.8 mm) operated at 50 °C, photodiode array detector operated at room temperature and 210 nm, and 0.005 mol/L H_2_SO_4_ as the mobile phase with a flow rate of 0.50 mL/min.

Excel 2010 was applied to perform an analysis of variance on experimental results by one-way ANOVA analysis as well as to determine the significance of citrate buffer concentration. The experimental results of fermentable sugars in hydrolysate, butanol concentration and cell growth in fermentation broth were selected, respectively. Significant variable effects were regarded if the reported *P* values were less than 0.05.

## Abbreviations

ABE: acetone–butanol–ethanol
VSVP: vapor stripping–vapor permeation
MAVS: membrane-assisted vapor stripping system
